# The use of autogenous tooth bone graft powder in the treatment of osseous defects after impacted mandibular third molar extraction: a prospective split-mouth clinical pilot study

**DOI:** 10.1186/s12903-022-02473-y

**Published:** 2022-10-02

**Authors:** Alimujiang Wushou, Yan Zheng, Yu Han, Zhi-cheng Yang, Fang-kai Han

**Affiliations:** 1 Department of Oral and Maxillofacial Surgery, Shanghai Stomatological Hospital and School of Stomatology, Fudan University, Shanghai, China; 2 Department of Oral and Maxillofacial Radiology, Shanghai Stomatological Hospital and School of Stomatology, Fudan University, Shanghai, China; 3 Shanghai Key Laboratory of Craniomaxillofacial Development and Diseases, Fudan University, Shanghai, China

**Keywords:** Impacted third molars, Autogenous tooth bone graft powder, Alveolar bone repair

## Abstract

**Background:**

Impaction of mandibular third molars (M3) is one of the most common diseases. Extraction of M3 usually exacerbates osseous defects at the distal aspect of the adjacent second molar (M2). BonMaker^®^ ATB has been cited as a novel autogenous bone grafting material. The aim of this pilot study was to introduce a novel method for repairing the distal osseous defects of M2 after the surgical removal of M3 with autogenous tooth graft powder (ATGP).

**Method:**

A total of five patients were enrolled in this prospective split-mouth clinical pilot study. Four impacted wisdom teeth were extracted bilaterally from each patient with proximal alveolar bone loss ≥ 5 mm of M3. The ATGP was prepared chairside from two extracted one side third molars and randomly implanted in one of the M3 extraction sockets, and the other side was treated with a blank and considered the control site. Patients were followed up at 6 months.

**Results:**

The five patients included three males and two females. Their ages ranged from 25 to 30 years, with a median of 27 years. Primary wound healing without complications was achieved in all the patients. There was a greater tendency for swelling of the cheeks and trismus to occur at the experimental site on the third postoperative day. Compared with the control site, the experimental site exhibited progressive bone filling and ossification in the sixth postoperative month. Moreover, the probing pocket depth of the experimental site was lower than that of the control site.

**Conclusion:**

The results of this study demonstrate that ATGP effectively and economically repairs distal osseous defects of M2. Further study is required to validate the effectiveness with a larger study population.

## Introduction

Impaction of mandibular third molars (M3) is one of the most common diseases, and the impaction rates range from 66 to 77% [1]. M3 generally gives rise to pericoronitis, maxillofacial space infection, odontogenic neoplastic changes, periodontitis, caries, and root resorption of adjacent second molars (M2) [2]. Extraction of M3 usually exacerbates alveolar bone resorption at the distal aspect of M2. Early studies showed that more than 40% of M3 cases presented probing pocket depth (PPD) ≥ 7 mm at the distal aspect of M2, and 33% of these study populations demonstrated worsening of the periodontal condition at the 2-year follow-up, with an increased PPD of at least 2 mm [3, 4]. Severe alveolar bone resorption eventually leads to the loss of M2. Therefore, early surgical removal of M3 plays an important role in the prevention of proximal alveolar bone loss [5].

Over the past three decades, extensive studies have investigated various methods to prevent and repair alveolar bone loss in order to improve the periodontal status of M2 after M3 extraction [6]. Compared with periodontal treatment and membrane placement, alveolar reconstructive procedures have demonstrated greater efficacy in inducing and accelerating bone regeneration. Bone graft substitutes include autogenous bone, mostly from mandible bone, allograft bone, synthetic bone and tissue engineered bone [7–10]. Platelet-rich plasma and platelet-rich fibrin combined with resorbable membranes have been shown to be effective alternative solutions [11, 12]. However, there is no therapeutic consensus algorithm. None of the existing repair methods are widely accepted and used as mainstream modalities in clinical practice because of their disadvantages, such as osteogenic instability, high cost and traumatic nature [6].

There is growing evidence for the use of autogenous tooth bone graft materials in alveolar bone reconstruction and bone augmentation, and this approach has achieved promising results [13–17]. Autogenous tooth bone graft materials have demonstrated the ideal characteristics of osteoconductivity, osteoinductivity, and osteogenicity, and these materials produce a nearly gold-standard graft and have low technique sensitivity [13, 18, 19]. BonMaker^®^ ATB is a novel autogenous bone grafting material produced by the mechanical and chemical processing of natural teeth [15]. The aim of this pilot study was to introduce a novel method for repairing the distal osseous defects of M2 after the surgical removal of M3 with ATGP.

## Materials and methods

### Study design

This is a prospective split-mouth small sample observational clinical pilot study. Four impacted wisdom teeth were extracted bilaterally from each patient with proximal alveolar bone loss ≥ 5 mm of M3. The ATGP was prepared onsite from two extracted one side third molars and randomly implanted in one of the M3 extraction sockets (experimental site), and the other side was treated with a blank and was considered the control site. Patients were followed up at six months. The institutional review board of Shanghai Stomatological Hospital & School, Fudan University, China, approved the study.

### Patients

From May 2021 to July 2021, five consecutive patients who had four third molars that needed to be extracted and were treated at the Department of Oral & Maxillofacial Surgery, Shanghai Stomatological Hospital and School, Fudan University, were enrolled. Inclusion criteria: (1) symmetrical proximal alveolar bone loss ≥ 5 mm of M3; (2) no history of systemic diseases, infectious diseases or genetic diseases; (3) no contraindications to conventional tooth extraction; (4) age of 25–30 years with good oral hygiene; (5) no history of drinking alcohol and smoking; and (6) no history of any other drug use. (7) Patients with proximal alveolar bone resorption of M3 found on oral examination and require M3 extraction. Exclusion Criteria: (1) M3 with severe pericoronitis and acute pulpitis that have not been effectively controlled; (2) pregnant or breastfeeding; and (3) M2 with periapical inflammation, crowding, ectasia, and torsion.

### Surgical procedure

**Fig. 1:**
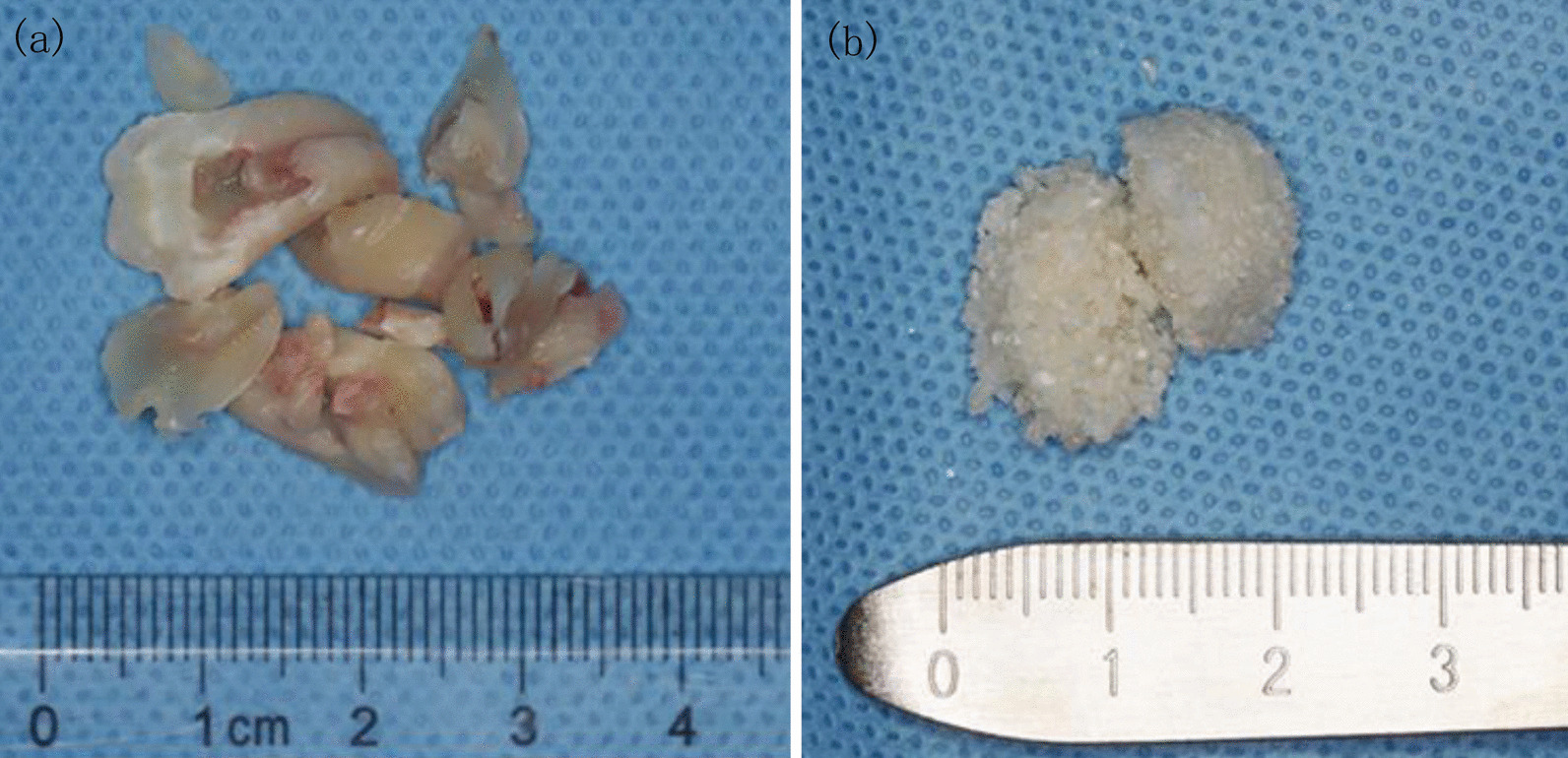
**a** Cleaned and dried extracted upper and lower third molar. **b** Autogenous tooth bone graft powder was prepared chair-side with the disinfected auto-tooth bone graft machine (BonMaker.^®^, Korea Dental Solutions Co. Ltd., South Korea)

The enrolled patients were informed of the details of the procedures and nature of the study itself, and then, the patients signed an informed consent form. Complete medical and dental histories were obtained, and preoperative radiographic evaluation was performed with panoramic radiographs and cone-beam computed tomography (CBCT). Surgical extraction and ATGP implantation were performed by two surgeons (W. A. & Y–Z. C.) under local anesthesia with articaine 4% and epinephrine 1:100,000 (Ultracain, Sanofi Aventis, Paris, France). A triangular flap was applied uniformly, and a full thickness mucoperiosteal flap was flipped to expose M3. Minimally invasive extraction, debridement and root planing were performed consecutively. The ATGP was prepared onsite as previously described according to the manufacturer’s instructions (Z. Y. & H. Y.) (Fig. 1) [15]. The M3 extraction socket was filled and compacted in layers with freshly prepared ATGP. After the placement of a rubber drainage strip, the mucoperiosteal flap was repositioned and closed tightly with interrupted sutures. Antibiotics were routinely used for one week after surgery combined with 10 mg prednisone for three days. Continuous icing was arranged for 48 h after surgery. Patients were re-evaluated one week postoperatively, and the sutures were removed. Postoperative follow-up exams were scheduled at six months (H.F-K.).

## Results

**Fig. 2:**
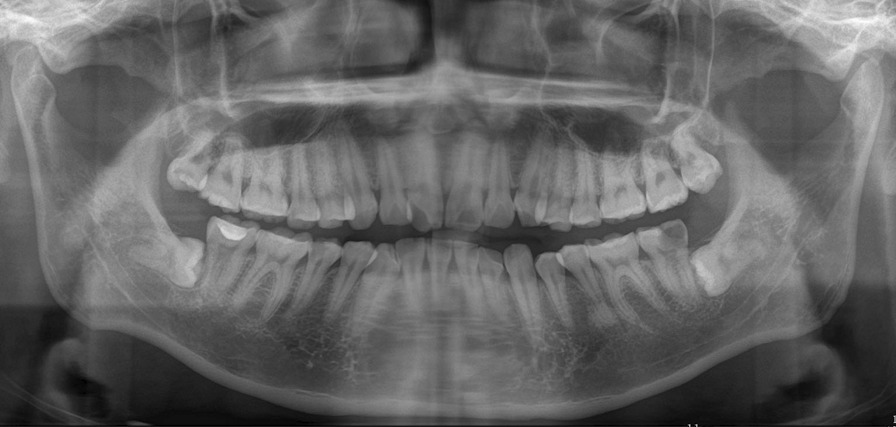
Preoperative panoramic radiographs

**Fig. 3:**
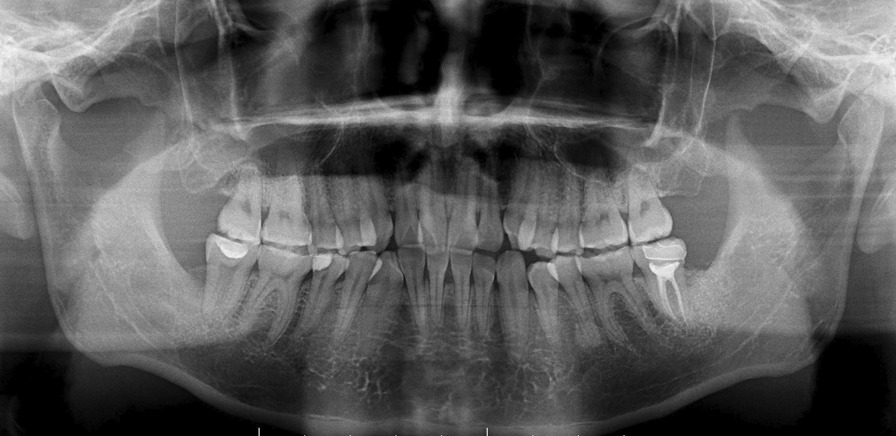
Postoperative panoramic radiographs

The five patients included three males and two females. Their ages ranged from 25 to 30 years, with a median of 27 years. The baseline characteristics of the included patients are presented in Table 1. Primary wound healing without complications was achieved in all the patients. There was a greater tendency for swelling of the cheeks and trismus to occur at the experimental site on the third postoperative day. None of the patients complained of any other pain or discomfort. Compared with the control site, the experimental site exhibited progressive bone filling and ossification in the sixth postoperative month. Moreover, the probing pocket depth of the experimental site was lower than that of the control site (Figs. 2, 3).

**Table 1:** Baseline characteristics of included patients

Patients	1	2	3	4	5
Age (years)	25	27	28	30	26
Gender	Male	Female	Female	Male	Male
*Location*					
Treatment	Left	Left	Right	Left	Right
Control	Right	Right	Left	Right	Left
*Pell–Gregory classification*					
Treatment	Class II, level B	Class I, level B	Class II, level B	Class II, level B	Class II, level B
Control	Class II, level A	Class I, level B	Class III, level B	Class II, level B	Class II, level C
*Winter classification*					
Treatment	Mesioangular	Mesioangular	Horizontal	Mesioangular	Mesioangular
Control	Horizontal	Mesioangular	Mesioangular	Mesioangular	Mesioangular
*Proximity to the mandibular canal*					
Treatment	No contact	Contact	No contact	No contact	No contact
Control	No contact	No contact	No contact	No contact	Contact
*Buccal–lingual classification*					
Treatment	Buccal	Central	Buccal	Central	Buccal
Control	Central	Central	Buccal	Buccal	Buccal
*PD (mm)*					
Treatment	3.0	3.3	3.2	3.1	3.2
Control	3.3	3.7	3.6	3.5	3.4

## Discussion

To date, many studies have confirmed that ATGP could be used as an alternative option, providing excellent biocompatibility without causing an immune response, contagion, or reaction to a foreign material in socket preservation, alveolar repair, and different kinds of bone augmentation in dental clinics [15, 20–27]. This study reached a similar conclusion. However, for the first time, we attempted to conduct a prospective clinical trial using ATGP prepared chairside from two extracted one side third molars to repair the distal osseous defects of M2. Within the investigation, ATGP is probably the most economical, convenient and effective bone substitute for repairing the distal osseous defects of M2 due to its rapid ossification, low absorption rate and good bone remodeling excellency.

Because of malposition and proximal alveolar bone loss, the M3 extraction sockets are usually larger than those of other teeth [1]. Physiological natural healing is often unsatisfactory. Due to the location, the self-cleaning of the M3 socket is relatively poor. If the wound is not closed, healing will be worse [2]. Therefore, two problems should be solved: first, the extraction socket should be filled, and second, the wound should be closed perfectly [6]. According to this study, the amount of ATGP prepared from two extracted third molars was exactly the amount needed to fill the M3 extraction socket. The distal osseous defect of M2 was repaired using graft power from a single extracted M3, and covering with a gelatin sponge also achieved a significant effect [28]. It could be inferred that spontaneous socket healing is the least common treatment option. Other advantages of ATGP are that no repairing periosteum is required [16, 18]. According to our observation, although the soft tissue swelling at the experimental site during the first three days after surgery was not mild, this process of ossification was occurred more quickly than the natural healing of the tooth extraction socket at the control site, and it occurred without any complications.

Autologous bone grafts remain the gold standard materials applied to optimize alveolar bone reconstruction and augmentation. However, the “rob Peter to pay Paul” supplying patterns are the greatest disadvantages of autologous bone grafts [10]. From a supply and demand perspective, extracted third molars are the best source for single tooth socket preservation and alveolar repair. If a patient has a smaller maxilla third molar or if the M3 was destroyed in the process of removal, insufficient amounts of ATGP would be obtained. When these cases occurred in this study, the root of M3 was filled with a gelatin sponge, the rest of the distal osseous defect of M2 was repaired with ATGP, and effective satisfactory results were also achieved. Bone resorption is another limitation of autologous bone grafts [29]. There have been no randomized controlled trials comparing autologous bone grafts with autogenous tooth bone grafts for the repair of alveolar bone defects. Regarding bone resorption, previous comparative single case studies have shown that autogenous tooth bone grafts are equivalent or superior to autologous bone grafts for repairing alveolar bone defects [13, 25, 30, 31].

## Conclusion

This is an observational pilot study, and the study results demonstrate that ATGP, prepared onsite, effectively, economically and conveniently repairs the distal osseous defects of M2. Although the limited sample size decreases the reliability of the study results, reports on the clinical application of ATGP made from two extracted third molars are very rare, and this investigation is clinically valuable. Larger case series or prospective clinical trials are required to validate the use of ATGP as a preferred and probably best alternative option for the treatment of osseous defects after the extraction of impacted mandibular third molars.

## Data Availability

All data generated or analyzed during this study are included in this published article.
